# Obstetrics and Diseases of Women and Children

**Published:** 1861-01

**Authors:** Wm. B. Atkinson

**Affiliations:** Lecturer on Obstetrics, and Diseases of Women and Children


### OBSTETRICS, AND DISEASES OF WOMEN AND CHILDREN.

BY WM. B. ATKINSON, M.D.,

LECTURER ON OBSTETRICS, AND DISEASES OF WOMEN AND CHILDREN.

I.—*Obstetric Statistics.* By T. T. Smart, L.R.C.P., of Edinburgh.

The number of cases was 5026. Face presentations, 7, born dead, 2; face to pubis, 20, dead, 6; hand with head, 3, dead, 1; shoulder, 3, all dead; hand and arm, 23, dead, 11; breech, 66, dead, 18; prolapsus of funis, 22, dead, 13; footling cases, 62, dead, 29; placenta prævia, 9, dead, 3, from exhaustion; forceps used in 32, dead, 13, all the mothers did well; craniotomy, 14; face presentation, 1; funis and vertex, 3; convulsions, 1; vertex, 9, three mothers died; puerperal convulsions, 18, died, 3; plurality of children, 72 cases, two distinct membranous sacs in each case, 16 distinct placentæ, 56 with but one placenta, all the females did well; hydatids, 1; acephaloid, 1; congenital encephalocele, 1 dead born; 1 case of deficiency of arm; imperforate anus, 1; hare-lip and cleft palate, 3; spina bifida, 3; os occipitis deficient in 1; frontal and part of parietal bones deficient in 1; deaths of mothers, 11.—(*Lancet*, Oct. 13, 1860.)

II.—*The Placenta and Membranes in Twin Pregnancies.* By Dr. SPAETH, of Vienna.

The statistics of Dr. Spaeth cover 126 cases, in 49 of which each child had a distinct placenta, chorion, and amnion; in 46, the placentæ were united, but the chorion and amnion of each were distinct; in 28 the placentæ were united, and but one chorion existed, but each had a separate amnion; in 2, the placentæ were united, and there was but one chorion and amnion. In cases of united placentæ, frequently the line of junction was indicated by a depression on the concave surface and by scanty deposits of fibrin. Even in cases of single chorion the dividing line was present. In no case where the chorion was single, was there the slightest indication of a fusion having occurred between two distinct membranes. At the level of the line which separated one amnion from the other, the chorion was always perfectly smooth, without thickening or depression. Where each chorion was distinct, but the placentæ united, the vessels were always independent and presented no anastomoses. But in two cases where the amnion was single, vascular communication existed between the two placentæ; in 17 of the 28 cases where a single chorion enveloped two amniotic membranes, vascular connection was found between the placentæ. These anastomoses were formed by large branches placed superficially on the inner face of the placenta. Hence, in the majority of cases where the placentæ are united, and where the chorion is single, there is free communication between the vascular system of the two placentæ. The anastomoses occur sometimes from vein to vein, and sometimes from vein to artery. In one case an anastomosis existed between an artery of one fœtus and a vein of another.—(*Zeitschrift der Gesellschaft der Aerzte zu Wien; Gaz. Hebdom.*, Aug. 24, 1860.)

III.—*Induction of Premature Labor.* By WM. THORN, M.D., of London.

Mrs. H., a very small woman, has been pregnant six times. Craniotomy was performed at term three times. The fourth pregnancy was terminated at seven months by rupturing the membranes, and giving a dose of ergot; but the child was born dead. Considering this result as due to the ergot, on the fifth occasion he brought on labor at the seventh month by the use alternately of the hot and cold douche, and rupturing the membranes. The child was quickly born and survived only a short time. Regarding the plans followed as wrong in some way, he endeavored so to induce the necessary premature delivery, that the process of natural labor might be imitated in its steady and gradual approach; his idea being that the deaths in the two former cases arose from the children being pressed too rapidly through a narrow and deformed pelvis. He, therefore, carefully removed, at about the seventh month, perhaps a week earlier, the plug from the os uteri, thus inducing a slight show. He then gave what he "knew to be a capital emmenagogue, viz., half a drachm of the biborate of soda, three times daily. At the end of sixteen days, no other action being perceptible except some slight uterine pains now and then, *the membranes ruptured spontaneously*, and the patient was easily delivered of a healthy male child, likely in every sense to do well." The only remarks he would make are, that he believes the satisfactory termination of the labor, and the safety of the child, are entirely due to the very gradual induction of the delivery, and that the biborate of soda fully answered his expectations in every respect.—(*Lancet*, Aug. 11, 1860.)

IV.—*Persulphate of Iron in Post-Partum Uterine Hemorrhage.* By G. Mendenhall, M.D., of Cincinnati.

In the case mentioned, all had gone well, when about ten minutes after delivery, the attention of the physician was attracted to the pallor of the patient, and the uterus being found distended, frictions were employed, which produced a temporary contraction. Ice was then used internally and externally, but the hemorrhage continued. Brandy and other means were employed as necessary, but four hours after delivery the hemorrhage continued steadily. The introduction of the hand rather increased it, and ergot was given with apparently little effect. A catheter was then introduced up to the fundus uteri, and about three ounces of the persulphate of iron (a saturated solution) were injected into the womb. This was retained as far as possible, no pain being produced, and the contraction was increased but slightly. Coagulation of the blood occurred in the uterus and vagina, and from that moment not another drop of fresh blood was discharged from either. Reaction came on, and the patient recovered without a bad symptom. For the next forty-eight hours the discharge was composed entirely of the disintegrated blood, which was succeeded by a serous, or sero-mucous discharge, colored by the salt, and which gradually became of the natural color of the lochia after the cessation of the presence of the red globules of the blood. It would seem to be applicable to post-partum hemorrhage where great relaxation of the uterus is present; in cases of hemorrhage accompanying abortion in the early months, and in excessive menorrhagia.—(*Cincinnati Lancet and Observer*, Nov. 1860.)

V.—*Rupture of the Uterus during Labor, in connection with Disease of the Cervix and Muscular Tissue.* By Edw. L. Ormerod, M.D.

The patient was thirty-six years of age, large and well made; had borne seven living and five dead children at term, and had also miscarried at the sixth month in the previous year. The first stage of her labors was always tedious. She had never suffered from uterine hemorrhage; it was not known whether she had habitual leucorrhœa, or had ever had syphilis. Three months prior to her death she was seized with seeming labor-pains, which continued severe for twenty-four hours, accompanied with a very free offensive discharge. This all ceased, and she went on well till a fortnight before her death. Labor then seemed to set in. She had a succession of regular pains, day and night, and at the outset an abundant discharge, which continued, though less in quantity. Nine hours before death the pains became more severe, and suddenly, after about three hours, she became collapsed, all uterine action ceased, and she died in an unconscious state. There was no external hemorrhage, upon examination. In the peritoneal cavity lay the child, a full-grown female, and the uterus was found to be torn straight down the posterior wall from fundus to cervix, the vagina not being implicated. The cervix was somewhat encysted, so that what seemed to be the os uteri was really a part of the canal of the cervix, occupied, in four-fifths of its circumference, by an elastic growth of a gelatinous appearance and studded with little bladders. The entrance to the uterus would not allow the passage of a cone of more than one inch and a half in diameter. The muscular substance of the uterus was soft and flabby. The pelvic dimensions were normal. There was no fatty degeneration anywhere.

A vertical section of the mass in the interior of the cervix showed a structure of a honey-comb appearance, from a number of irregularly cylindrical cells, distended with a clear mucus. The whole mass was nearly three-quarters of an inch thick, and the bladders seen were the protruding ends of some of these dilated mucous follicles.

The disease seems to have been simple hypertrophy of the glands of the cervix, with fibrous degeneration of the adjacent muscular structure. It was not the bulk of the tumor which caused the obstruction, but the rigidity of the tissues about the orifice of the womb. This condition had apparently existed several years, increasing till the rupture occurred by the fruitless efforts to overcome the resistance. When did the rupture occur; and how? It would seem to have been gradual, for it is inconceivable that such an injury, and so much hemorrhage, (five pints of blood being found in the peritoneal cavity,) could occur suddenly without proving immediately fatal.—(*Lancet*, September 29, 1860.)

### VI.—*Nineteen New Cases of Eclampsia of Pregnancy, Labor, and Puerpery, and its connection with Bright's Disease.* By DR. KRASSING.

Dr. Krassing gives the following summary:—

Of the patients, 11 were from 18 to 25 years of age, 5 from 26 to 38.

Eclampsia occurred in the proportion of 1 in 500 labors. Primiparæ were more frequently attacked.

There were two cases of twins.

The attacks came on mostly without premonitory symptoms; ten times during dilatation of the os uteri; once during an attempt at turning, the os being dilated; five times during the puerperal period—three times on the first, one on the second, and one on the eighth day.

In the 10 cases occurring during dilatation, pregnancy had reached its normal term in 4; the eighth month in 4; the ninth in 2. The others occurred at the regular period of labor.

The number of attacks ranged from 1 to 81, and the period of pregnancy or of puerpery had no influence on the frequency.

The duration of the individual attacks varied from 1 to 3 minutes, only seldom attaining 10 minutes. Premonitory symptoms appeared in 6.

In one case only was albumen absent from the urine. In all the rest there was evidence of Bright's disease. Œdematous infiltration occurred 10 times. In one case without œdema, there occurred 81 fits; in one without albuminuria, 60 fits.

Seven recovered, 9 died. Of the recoveries, 4 had convulsions during labor, 3 during puerpery. Death happened twice during convulsions, five times during coma, and twice from after-diseases.

Dissection of 9 cases exhibited: anæmia and serous soaking of the brain, 6 times; hyperæmia, 1; intermeningeal apoplexy, 1; nothing abnormal in brain in 1. Hyperæmia and inflammatory exudation in kidneys, 3 times; fatty degeneration, 4; atrophy, 1; no disease of the kidneys, 1; œdema of lungs, 4; peritonitis, 2; stenosis of the right ureter, 2; tubercles in lungs, 1.

One breech, and the rest head presentations. Of the 18 children, 10 were boys, 8 girls; 15 born alive, 3 dead.

*Treatment.*—There were 5 bleedings to the extent of 10 ounces; 3 of these ended fatally, and in one there was a manifest increase of the attacks. Chloroform, inhaled in 6, mostly moderated the attacks. Opiates were given by the mouth and rectum 10 times, in large doses, until the accession of sleep, and did good service. To excite diuresis, lemon-juice, tartaric acid, and gum benzoin were given. Cold wraps and douches were applied to the head.

Forced delivery was not resorted to; but of the 10 cases of the dilatation period, the labor in 4 was hastened by the introduction of a flexible catheter; in 1, colpeurysis; in 4, dilatation with the fingers; in 6, the forceps; in 1, turning; in 1, perforation; in 1, the Cæsarean section was employed during life.—(*Monatschr. f. Geburtsk.*, Mai, 1860; *Brit. and. For. Med.-Chir. Rev.*)

VII.—*On a Puerperal Epidemic at the Wurzburg Obstetrical Establishment.* By DR. O. VON FRANQUE.

This institution, which is under the care of Scanzoni, has been but recently established, and is placed in a healthy, airy position. It averages 360 pregnant women, and from 350 to 360 births annually. The epidemic occurred during February, March, and April, 1859. In this period there were 99 deliveries; the forceps were applied 4 times, and turning was resorted to once. 30 of these cases were attacked with puerperal fever, and 9 died. One of the women died of phthisis, and one of eclampsia. Of the 102 children born, 8 were dead, and 9 died subsequently. Premonitions of the epidemic were met with at the close of 1858, in the form of mild peritonitis, etc. About this time, irregularities in the parturient process were frequent, consisting in deficiency of pains, irregular spasmodic contractions, and rigidity of the os uteri. In some of the fatal cases this last condition caused excessive prolongation of the labor, which itself powerfully predisposes to the disease. Almost all had more or less considerable hemorrhage, the uterus remaining soft and large, showing no disposition to contract.

Puerperal affections exhibit themselves under two principal forms, viz., with hyperinosis of the blood, and with primary dissolution of that fluid. The latter form was only observed in any considerable degree in two cases, which were very acute—both fatal. It is remarkable that the most acute of all the cases, in which death occurred within twenty hours after delivery, was seized in April, when the epidemic was declining. The cases connected with a hyperinotic state of the blood were less rapid. The first appearances of disease were manifested on from the second to the fifth day, commencing either in the form of a localized endometritis or peritonitis, or of the two together. There were 28 cases of this form—7 proved fatal. In one of the 7, puerperal mania occurred.

The treatment consisted in local cataplasms, mercurial frictions, warm baths, and small doses of calomel and opium. The post-mortem appearances were a large relaxed uterus, with the cervical portion softened, and its inner surface lined with diphtheritic or gangrenous deposit; fibro-purulent exudations in the abdominal cavity; enlarged spleen; and a dark, fluid blood in all the veins, the heart, and the cerebral sinuses, exhibiting, hence, the signs of a dissolved condition of the blood, the result of the continuation of the diseased process.

In the milder cases cataplasms only were employed, and phosphoric acid given with the beverages; local bleeding, and especially warm baths, being resorted to only when the local pain proved excessive. In the majority, ten to twelve days sufficed for the treatment. There were also cases of febrile action without special local manifestation, and others in which, with a moderately rapid pulse, more or less prostration and malaise, there was an abnormal enlargement of the abdomen without pain on pressure. The involution of the uterus was performed with remarkable slowness. All these cases recovered.

One remarkable fact is, that in certain cases of labor, during the height of the epidemic, where, on account of difficulty and delay, or exhaustion, the worst prognosis was given, no ill effects resulted. In fact, during such an epidemic, individuals not predisposed to puerperal disease, may undergo most terrible labors without being attacked, while others, with a normal labor, fall victims to the disease.

The influence extended to the fœtus and child; all who were born dead, or died soon, exhibited a diseased condition of the blood, of which they had become the subject while *in utero*. The blood was dark and fluid, the spleen enlarged, and the umbilical arteries often contained pus.

No other cause could be assigned for the epidemic than the prevalence of certain atmospheric influences. Miasmatic influences could not have been generated within the walls of an establishment so newly built and well contrived, and not overcrowded. Moreover, puerperal diseases prevailed at the same time in Wurzburg and its vicinity, which were not, it is of importance to observe, treated by the physicians of this institution. To this may be added the greater prevalence of hemorrhages, and the greater mortality from puerperal diseases at this time. An influence which has often proved very mischievous in lying-in-hospitals during epidemics, viz., the presence of numerous male individuals, did not come into operation here. Individuality, too, exerted no influence; for the feeble and strong were alike attacked. In fact, the fatal cases occurred among the most strong and powerful women, while the feeble suffered comparatively little.—(*Scanzoni's Beitrage*, band iv. pp. 238–249; *Med. Times and Gazette.*)

### VIII.—*Creosote Vaginal Injections in certain forms of Puerperal Fever.* By Dr. MACKENZIE, of London.

For a number of years, Dr. Mackenzie has employed injections of creosote of the strength of ♏viij—xij to the pint of mucilage, in that form of puerperal fever which originates from the absorption of morbid secretions from the vagina, etc. He quotes a case where delivery was effected by destruction of the child, after which all seemed well; but the next day the abdomen was found to be swollen, and there was much pain over its whole surface. A dose of castor-oil and turpentine was given, and followed by a mixture of tinc. opii, gtt. v, with ammoniæ sesquicarb. gr. v, every four hours, and an injection of creosote was thrown into the vagina. The day following, but slight improvement being manifested, a saline antimonial mixture was given in place of the opium and ammonia, and warm fomentations were applied over the abdomen; the injection having been repeated, another day brought a change for the better, the treatment being continued; and in the course of some two weeks she was discharged from the hospital perfectly well.—(*Brit. Med. Jour.*, March 3, 1860.)

IX.—*On the Continuance of Ovulation during Pregnancy.* By Prof. SCANZONI.

Prof. Scanzoni enters into some speculations concerning the continuance of ovulation during pregnancy. His opinions have been recently contested by Kussmaul. Scanzoni argues, that since the bursting of the Graafian vesicle and its consequences are entirely the result of the menstrual hyperæmia of the ovarium, and that the latter can differ greatly, the conclusion must be granted that the menstrual prolegomena are not always or necessarily connected with the bursting of the follicle which embraces a ripe ovum. He reasons ingeniously to prove that although he is unable to show fresh-burst Graafian vesicles in pregnant women, yet there are strong grounds which render it more than probable that there persists a periodical ripening of ova during pregnancy, although without bursting of the follicle. But the reasoning is essentially speculative, and takes too little account of opposing facts.—(*Beitrage*, band iv., 1860; *Brit. and For. Med.-Chir. Rev.*)

X.—1. *Treatment of Menorrhagia.* By GEO. S. MORRIS, M.R.C.S.

In answer to an inquiry concerning the treatment of menorrhagia, where the usual remedies, viz., ergot, steel, quinia, and rest, have entirely failed, Dr. Morris recommends very strongly the use of gallic acid in five-grain doses three times a day. With this article he has never failed in curing his patient.

2. *Ibid.* By R. B. NEWHOUSE, M.R.C.S.

Dr. Newhouse makes a similar suggestion, urging the use of the gallic acid in the same dose during the menstrual flow, and between the periods small doses of nux vomica, strychnia, or quinia with hydrochloric acid.—(*Lancet*, Nov. 1860.)

XI.—*Cancer of the Uterus.* By M. ARAN, of Paris.

*Deep-seated* cancer of the uterus is situated in the cellular tissue of the fibres of that organ. In any form, it makes for itself a cavity by displacing the neighboring muscular fibres, which, with the mucous membrane, are generally healthy. Soon new deposits occur, and then these tissues become atrophied and disappear. The morbid matter softens, the tissues ulcerate, and the hardness and projections are replaced by irregular excavations with jagged edges, and bathed by a sanious fetid fluid.

*Superficial or mucous* cancer commences with hypertrophy of the papillæ and follicles of the mucous membrane; but we may have it either continuing thus as a *vegetating* cancer, or in other cases it may become of the *ulcerating* variety. It first appears most generally in the form of very fine mammillated projections, like shot or sand, which may be either hard or soft. The epithelium is not destroyed, and in some cases it forms foliated lamellæ separated by furrows. In others the epithelium may accumulate and form polypiform tumors. In this, especially, vascularity abounds, causing frequent and profuse hemorrhage which soon exhausts the patient.

The *ulcerated* form may be circumscribed or diffused. It is rarely primitive, most commonly following the other forms. Even in the vegetative form, when it appears to have an outward tendency, the cancer extends into the subjacent tissue by the multiplication of epithelial elements, and also determines the deposition of epithelial tumors in the subperitoneal cellular tissue, or in the uterine appendages, as well as in the vagina, bladder, and rectum. When ulceration of the surface of the cancer has commenced, a similar process is set up in the more deeply-seated and distant deposits, and fistulous communications are formed between the different organs.

These two forms of cancer do not possess the same amount of danger. The deep-seated is more grave, in infecting the system more rapidly and being less accessible to local remedies. The superficial may be advantageously treated by caustics. One case was thus cured five or six years ago, and is yet living. Death occurs less frequently than when other organs are affected; the most common causes are peritonitis, inflammation of the psoæ muscles, or phlegmasia alba from obliteration of the veins. In some cases the uterus may be compressed by the tumor, and dilatation occur above the point of pressure; hydronephrosis or distention of the kidney by urine takes place, resulting in uræmic phenomena.—(*Gaz. des Hôpitaux*, Sept. 11 and 13; *Brit. Med. Jour.*)

### XII.—*Treatment of Inflammatory Affections of the Female Breast.* By W. H. BYFORD, M.D., of Chicago.

After fully describing the character, of these affections, Professor Byford comes to their treatment. He arranges that for inflammation of the nipples under the heads of prophylactic, palliative, and curative. The nipple must be prepared for its duties. The causes operating upon it produce abrasions, and their action is facilitated by the natural and acquired tenderness of the structures, particularly the epidermis and skin. Hence these must be hardened. The nipple should be covered lightly during pregnancy and nursing; the thinner and more permeable the covering the better. It should freely admit the air. At the same time the organ "*should be subjected pretty constantly to moderately rough friction.*"

An excellent dressing for the nipples for the last two months, is a rough, coarse sponge, so cut as to cover the areola; surround and cover loosely, but touch every part of the nipple. Over this there should be but one thickness of clothing, so as to allow of the evaporation of fluid as fast as secreted, and the free admission of air. In cold weather, of course, the parts should be covered more when going out. The nipples should be occasionally moistened with water, and allowed to dry slowly; friction with a dry towel or the fingers will assist.

During lactation, the same rules should be observed, and after nursing, the nipple should be wiped clean and dry before being covered. A little glycerin or olive oil will prevent cracking. When inflammation comes on, palliatives and curative measures are demanded. The healing process being continually interrupted by the performance of the functions of the organ, it is necessary to protect the part from the effect of these interruptions.

Artificial means are required, which intervene between the mouth of the child and the nipple. For this purpose the shield must be employed. This should be made in the form of a conical hat, having a rim, a crown cavity, with a draught tube rising out of the top for the passage of the milk. This rim should be large enough to cover the areola, the crown passing over the nipple, merely touching it on the sides. If the abrasions are on the summit of the nipple, the shield should be so deep that, when drawn, the top of the organ will not touch, or else it will cause pain. But if the cracks are on the side or base of the organ, then the cavity of the shield must be shallow, so that the top of the nipple touches its bottom in such a manner as to prevent any stretching, and to bring the pressure entirely on the top. In this latter case, the bottom of the cavity should be as smooth as possible, and correspond in shape to the summit of the nipple, in order to prevent unequal pressure. A soft linen rag, properly adjusted over the draught tube, is preferable to any other envelope.

M. Legroux mentions the following ingenious contrivance. He applies this mixture:—

℞.—Collodion, p. xxx;

Ol. Ricini, p. ss;

Ol. Terebinth. p. jss.

This is quite adhesive, and dries less quickly than collodion, on the areola with a brush, so as to encircle, but not touch, the nipple for the width of an inch. While yet soft the nipple is covered with gold-beater's skin, which is pressed well down upon the mixture. Thus is formed a smooth and pliant covering. Holes are pricked through the skin with a needle, to allow of the passage of the milk. Before sucking, this must be moistened with sugar and milk.

The curative means for sore nipples are various. The same will not do for abrasions as well as ulcerations. Nature is to be imitated by forming a cuticle for the part.

Abrasions may be covered with starch and mucilage. The following is a good mixture:—

℞.—Cerat. Alb. ℥ij;

Ol. Amyg. Dulc. ʒj;

Mel. Despum. ℥ss. M.

Dissolve with gentle heat, and add Bals. Canad. ʒijss.

Apply each time of nursing. When the cracks are deep, close them by pressing their edges together, and covering with collodion in a thick and wide coat; this must be renewed when found necessary. When ulceration exists, it will be acute or chronic. Act as for this affection elsewhere; deplete, if acute, by leeches, and apply cold emollient poultices; or envelop the nipple in a thin layer of thick mucilage, covered by oil-silk, so as neatly to fit the organ, kept cold by ice applied in a bladder. When these remedies are not necessary, apply mucilaginous and bland ointment applications. Alum and tannin are good at first; sulphate of zinc and borax come next in respect to time. One scruple of tannin to one ounce of rose-water, five grains of alum, or sulphate of zinc are useful in the early stages, when the acute symptoms are subdued.

The following are useful:—

℞.—Sodæ Subborat. ℨss;

Glycerin, ℨij;

Aq. Rosar. f℥jss.

M. Use as a wash after sucking.

℞.—Sodæ Subborat. ℨij;

Cretæ Præp. ℥j;

Spt. Vini,

Aq. Rosar. āā f℥iij.

Mix and dissolve.

The latter may be used when the ulcer is becoming indolent. In the chronic form strong astringents and stimulants become necessary. A skillful use of the sulphate of copper and nitrate of silver will shorten the course of these ulcers. The latter applied solid to the surface, not oftener than once in eight days, is excellent. In the interval the sore may be dressed with tannin or alum in solution, When irritable, an ointment may be used, made of belladonna, hyoscyamus, or opium. One very good expedient, which will often entirely change the character of the ulcer, is to anæsthetize the part with ice, as practiced prior to operating.

When the lymphatic glands become affected, antiphlogistic measures must be employed; and when chronic, alteratives, tonics, liniments, etc., according to the peculiarities of the case. The treatment of milk abscess is of great importance. It should be prevented if possible, by proper management at the outset. When the nipple is deficient, or, from any cause, prehension is deemed impossible, it is decidedly improper to attempt nursing. In other cases, prolonged and judicious efforts should be made to render the organ useful. The first thing is to take perpendicular pressure off the top of the nipple, by some device to prevent the dress from forcing it in, and this, if possible, should be commenced early in pregnancy. For this purpose a shield should be employed, which will cause a pitting of the anterior surface of the breast, and a projection of the nipple. When called upon to treat a rudimentary nipple, after parturition, the effect must be more prompt. In many cases the organ may be made available by causing it to erect itself by simple titillation by the finger, and immediately applying the child; or by placing a thick layer of collodion, around it on the areola, which, drying, elevates the nipple. Then, by keeping the reservoirs empty, abscess is prevented. To aid in this we have various tubes and pumps, but all of which are objectionable. A puppy is often used, but it likewise is liable to irritate and excoriate the nipple.

The only proper way is by the mouth of an adult, varying the pressure or force to suit the tenderness of the part.

A very useful class of measures are those to suppress the secretion, and thus relieve the distention, as opium in large doses, or applied as an ointment; but belladonna seems to have acquired most renown. Numerous instances are reported of its great value in such cases. Much depends upon its strength and application by inunction till the production of its characteristic effects upon the system. Cold, as a local remedy, is beneficial. The temperature of the breasts for this purpose should be kept steadily at about 40 or 45 degrees, as by water running through an india-rubber bag enveloping the organ, or the application of a bladder. No bad effects are to be apprehended from it. Internally, a saline cathartic may be given every other day, and two grains of iodide of potassium every four hours will materially assist.

Acute inflammation, the effect of congestion, is apt to be extensive, and will require energetic treatment. Warm fomentations may be applied for the first few hours with the hope of establishing the secretion of milk. A decided venesection will often turn the balance in favor of resolution. Immediately after this, the use of the veratrum viride may be commenced in doses of six drops every four hours, till the pulse is brought down below the normal standard, and then kept there. One grain of calomel, with a quarter of a grain of sulphate of morphia, may be given, if the pain is urgent, say every four or six hours. A lotion of one part of sulphuric ether to two parts of alcohol will be a good soothing adjunct, after the inflammation becomes permanent. These measures should not be abandoned for warm poultices until suppuration is clearly evident, by which plan we may often limit the extent of this process. In this state of the gland, the most moderate means only should be employed to draw the breast. Retained milk is not the cause of inflammation here, as in milk abscess. If glandular inflammation is complicated with that of the reservoirs, the treatment for both must be combined, as local and general antiphlogistics with means to arrest the secretion and empty the reservoirs. Chronic inflammation will be cured by treatment similar to that for other glandular inflammations, as leeches, mercurials, iodine, and vegetable alteratives, internally and externally. Much reliance can be placed upon well-regulated and graduated pressure with adhesive straps, pressing the diseased part against the ribs; or with collodion thoroughly incasing the breast. When pus forms, evacuate it early, though where the abscess is deep, it is desirable to wait until the pressure from within has caused condensation of the overlying tissues, otherwise a large opening will be required. In milk abscess the earlier and smaller the opening the better. The effect of suppuration and evacuation of a milk reservoir is often to destroy its cavity, but in some cases a milk fistula is formed. This may be closed by an occasional application of the nitrate of silver. Worse than these are the tortuous lacunæ, that sometimes result from the deep glandular abscess of the breast, and which are generally very difficult to cure. Injection of iodine is most to be relied upon. This may be done by inserting a soft, flexible catheter to the bottom of the canal and throwing the injection through it so as to apply it without dilution to the bottom of the fistula. This favors the shallowing instead of the narrowing of the cavity. Of course it is never advisable to slit up these obstinate puriferous ducts, because of the amount of tissue that might be damaged, which it is desirable to save.—(*Chicago Med. Exam.*, Sept. 1860.)

XIII.—*Urticaria as a Symptom of Irritation of the Female Sexual Organs.* By PROF. SCANZONI.

Prof. Scanzoni observes that though it has been long known that chronic affections of the female sexual organs are not seldom accompanied by skin diseases, yet the influence of a more sudden irritation of these organs upon the cutaneous surface is not so well established. To render this more positive, he relates a number of cases. A lady, aged thirty-four, was ordered a slight leeching of the vaginal portion of the cervix uteri for a chronic metritis. In ten minutes after the application had been made, the patient was attacked with violent fever and slight delirium. In half an hour her skin was found to be of a scarlet red, and the next day numerous urticaria appeared over her whole body. Two days after, the eruption disappeared and desquamation took place. On four different occasions the leeches were applied as before, without this result, but on the fifth, a similar train of symptoms followed, and that so rapidly, that no room was left for doubt as to cause and effect. A second case, aged twenty-eight, had chronic uterine infarctus; leeches were applied to the cervix; they had scarcely taken hold before she was attacked with violent abdominal pains, fever, and a profuse eruption of urticaria. A third case occurred in precisely the same manner. These cases admit of no other explanation than that the irritation of the uterine nerves from the bites of the leeches caused an unusual disturbance of the vascular system, which gave rise to the eruption. In proof that these phenomena were not a consequence of any poison conveyed by the leeches, it is to be observed that such symptoms never result from the application of leeches to other regions, while even very slight irritation of the sexual organs, as by the finger or speculum, or caustic, will often produce erythema of the face, neck, breast, etc., which disappears as rapidly as it comes on.—(*Wurzburger Medicin. Zeitschrift*, band i.; *Med. Gaz. and Times.*)

XIV.—*Affections of the Hip-Joint in Consequence of Uterine Disease.* By DR. HOPPE.

Two cases are reported, one unmarried, forty-five years of age, the other a widow, aged thirty-six, both of whom had been suffering for a long time from a disease of the womb and an affection of the hip-joint. The symptoms of the latter malady are: 1st. Pains which occupy the whole of the thigh, especially in its anterior and exterior aspect, or only in the circumference of the trochanter; they are very intense, lasting through day and night, with very short intermissions. 2d. Contraction of the adductor muscles and of some of the flexors. Both symptoms are referable not so much to an affection of the joint itself as to an irritation in its neighborhood; this becomes more likely by the fact that there exists no deformity about the nates. As a further proof that the named affection was owing to a disease of the womb, Dr. Hoppe remarks that he has frequently observed painful sensations around the trochanter in connection with uterine disease. These patients complained of a sensation of pressure and burning in the region of the trochanters, which diminished gradually, and finally disappeared under a treatment adapted to the disease of the womb. The author points to a fact more generally known, viz., the occurrence of gonalgia as a consequence of affection of the womb.—(*Pr. Ver. Ztg.*, N. F. iii. 2, 1860; *Amer. Med. Times.*)

XV.—*Shortness of the Umbilical Cord.* By M. Devillers, of Paris.

M. Devillers, in his lecture on this subject before the Academy of Medicine, offers the following signs as indicating this condition prior to delivery:—

A sudden decrease or cessation of the motions of the fœtus at a period approaching the end of gestation, in accidental shortening or less extended movements during a great part of pregnancy, and especially near its termination in natural shortening.

Occasional premature uterine pains.

Continued elevation of the fundus of the uterus at the moment of labor, and in females in whom the pelvis is well formed, and the infant presents in a normal manner.

During the whole labor, a tension, a rigidity of the walls of the uterus, even between the pains, and until the expulsion of the fœtus. Sometimes a pain felt at a fixed point in the fundus at the moment of contraction. The presence of the umbilical sound on one or more points of the surface of the uterus, above all, after the rupture of the membranes, but only in cases of accidental shortening.

Often a slow progress of the labor with successive feeble uterine contractions in the case of very decided shortening, without any other apparent cause. Terminal pains very perceptible, and as if repressed in the last moments of labor.

Signs of suffering of the fœtus at an advanced period of labor, especially when the parts of the fœtus are deeply engaged in the pelvis. Often a quick termination of the accouchement, and preceded or accompanied by a slight hemorrhage.

The danger of the natural shortening does not become apparent until the close of labor; while that of the accidental form is seen sooner, and arises principally from compression of the cord.

Finally, compression is a cause of danger to the infant more frequent than is generally believed.

Relative to the treatment, M. Devillers recommends frictions with belladonna to the neck of the uterus, with the design of modifying the resistance of the walls and orifice of the uterus; and also that the physician may seek to disengage or relax the cord; or, if that is not practicable, to cut it, and bruise the fœtal extremity, and finish the accouchement as quickly as possible. He rejects *version* as irrational and dangerous, and prefers the forceps.—(*Gazette Médicale*, Nov. 1860.)

